# Pneumococcal and influenza vaccination rates and their determinants in children with chronic medical conditions

**DOI:** 10.1186/1824-7288-36-28

**Published:** 2010-03-26

**Authors:** Antonietta Giannattasio, Veronica Squeglia, Andrea Lo Vecchio, Maria Teresa Russo, Alessandro Barbarino, Raffaella Carlomagno, Alfredo Guarino

**Affiliations:** 1 Department of Paediatrics, University Federico II, via S. Pansini 5, 80131, Naples, Italy

## Abstract

**Background:**

To investigate the rates of pneumococcal and influenza vaccinations and their determinants in children with chronic medical conditions.

**Patients and Methods:**

Children with HIV infection, cystic fibrosis, liver transplantation and diabetes mellitus were enrolled. Physicians of regional Reference Centres for each condition, primary care paediatricians and caregivers of children provided information through specific questionnaires. For diabetes, 3 Reference Centres were included.

**Results:**

Less than 25% of children in each group received pneumococcal vaccination. Vaccination rates against influenza were 73% in patients with HIV-infection, 90% in patients with cystic fibrosis, 76% in patients with liver transplantation, and ranged from 21% to 61% in patients with diabetes mellitus. Reference Centres rather than primary care paediatricians had a major role in promoting vaccinations. Lack of information was the main reason for missing vaccination. Awareness of the severity of pneumococcus infection by key informants of at-risk children was associated with higher vaccination rate.

**Conclusions:**

Vaccination rates in children with chronic conditions were poor for pneumococcus and slightly better for influenza. Barriers to vaccination include lack of awareness, health care and organization problems.

## Background

Among vaccine preventable diseases, two are of outstanding importance and provide an interesting area of evaluation: pneumococcus and influenza infections. The first is a relatively common and potentially severe infection for which vaccines are widely available, effective and relatively widespread. Influenza has peculiar features related to variable degree and the need of annual vaccination.

Subjects with chronic medical conditions are at increased risk for severe complications related to vaccine-preventable infections and should be extensively immunized [1,2]. Influenza and invasive Streptococcus pneumoniae infections are associated with increased morbidity, hospitalisation and mortality in children with chronic medical conditions compared to healthy children [3-7].

Criteria for influenza immunization in USA by Advisory Committee for Immunization Practice (ACIP) now include all children aged 6 months to 18 years [8]. In Italy, influenza vaccination is not routinely offered to healthy children, whereas it is recommended and offered without costs to children with chronic conditions. The same is true for pneumococcal vaccination [9].

Despite long-standing recommendations to provide pneumococcal and annual influenza vaccinations to children with chronic medical conditions, immunization rates in these vulnerable populations are poor [10,11]. A recent cluster survey performed in Italy showed less than 10% coverage for influenza vaccination and a variable rate for pneumococcus vaccination (49% in at-risk children aged less than 24 months and less than 15% in adolescents) [12].

Several factors hamper implementation of these vaccinations, including problems in identifying at risk children and limited awareness of specific recommendations [13,14]. Vaccination of at-risk children is largely a matter of organisation. In Italy, as well as in many other western Countries, children with chronic conditions are generally seen at specific Reference Centres, often located in Universities or major Children's Hospitals. However, children with chronic diseases are seen also by primary care paediatricians (or family paediatricians), like all children in the Italian Health Service. Moreover, vaccination centres provide vaccinations to both healthy and at-risk children. Therefore, there are multiple services through which a child with a chronic disease can receive immunization.

The aims of the present study were to investigate the rates and the correlates of pneumococcal and influenza vaccinations in a large cohort of at-risk children. Four chronic medical conditions were selected in order to analyse different models of immunization.

## Methods

### Study populations

Children aged 2-18 years with the following conditions were included: HIV infection (HIV), cystic fibrosis (CF), type I diabetes mellitus (DM) and children who received liver transplantation (LTx). Children <2 years and those diagnosed as having any of the selected chronic conditions since less than 1 year were excluded, in order to evaluate the rates for influenza vaccination in two subsequent seasons.

Three hundred and forty-three high-risk children (174 males; median age 13 years, range 2-18) were enrolled, including 40 patients with HIV, 39 with CF, 59 with LTx and 205 with DM. The demographic features were similar in all 4 groups (table 1).

**Table 1:** Demographic features of 343 at-risk children

Patients	HIV	CF	LTx	DM
Number	40	39	59	205
Males	17 (43%)	18 (46%)	34 (58%)	105 (51%)
Median age in years (range)	11 (2-18)	11 (2-18)	9.5 (2-18)	13 (2-18)
Median age at diagnosis in years (range)	3 (1-12)	0.3 (0.1-15)	0.1 (0.1-13)	8 (2-16)

*HIV*, human immunodeficiency virus infection; *CF*, cystic fibrosis; *DM*, type I diabetes mellitus; *LTx*, liver transplantation.

Physicians involved in the management of children with chronic diseases, including paediatricians of the Reference Centres and primary care paediatricians, were enrolled and received a specific questionnaire. The study was performed in accordance with the Declaration of Helsinki. Parents or legal guardians of children received a specific questionnaire providing that informed consent had been given.

### Data sources

Patients with chronic medical conditions were identified through the Regional Reference Centres for each disease. The Reference Centres for HIV infection, for CF and for LTx (Department of Paediatrics, University Federico II) and all three Reference Centres for DM located in Campania Region (University Federico II, Second University and San Sebastiano Hospital of Caserta) took part into the study.

### Key instrument

A questionnaire was administered face-to-face to caregivers of enrolled subjects with high-risk medical conditions during their routine clinical evaluation at the Reference Centre. Parental report of vaccination status has been previously validated and has a reasonable sensitivity, specificity and reliability [15,16]. The questionnaire included a demographic section with age, sex and age at diagnosis, questions to investigate caregivers' awareness about infection, their children's vaccination status for pneumococcus and influenza, and factors that might have affected vaccination status. Children were considered immunized against pneumococcus if they have received heptavalent pneumococcal conjugate vaccine or pneumococcal polysaccharide vaccine, according to their age. Inactivated influenza vaccine is the only vaccine available in Italy.

In addition, a call to participate to the study was sent by e-mail to all members of Italian Society of Paediatrics of Campania region. One hundred and thirteen primary care paediatricians, corresponding to approximately 15% of the total number of primary care paediatricians of Campania Region, accepted to participate to the study and received a specific questionnaire on pneumococcal and influenza diseases. Items included the specific risks of pneumococcal and influenza infections in children with chronic medical conditions, knowledge of current national recommendations on vaccination and the number of patients with high-risk conditions routinely seen at their practice. Finally, physicians of Reference Centres were interviewed in order to investigate whether they routinely recommend pneumococcal and influenza vaccinations to at-risk children.

### Data analysis

Data are expressed as medians and ranges or means and standard deviations (SD). The chi square and the *t *tests for independent samples were used to compare categorical and continuous variables, respectively. The Mann-Whitney test was used to compare non parametric data. A p value < 0.05 was considered statistically significant.

## Results

### Pneumococcal and influenza vaccination rates

**Figure 1:**
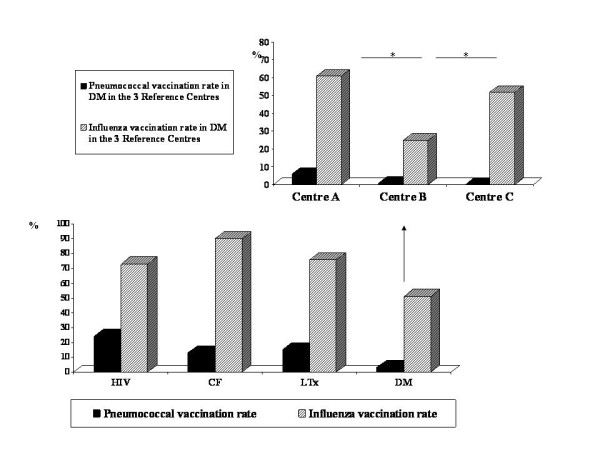
**Pneumococcal and influenza vaccination rates in 343 high-risk children**. Inset: percentage of patients with DM vaccinated for pneumococcus and influenza seen at each of the three Reference Centres (*p < 0.05). The lowest rate of pneumococcal and influenza vaccinations were obtained in patients with DM (p < 0.05 in all cases).

Vaccination rates against pneumococcus were low and largely below 50% in all high-risk categories, the lowest being in subjects with DM (DM versus HIV: p = 0.0007; DM versus CF: p = 0.02; DM versus LTx: p = 0.002) (figure 1). Vaccinated and not vaccinated children did not differ by sex (17/29 males versus 155/314, respectively; p > 0.05) or mean duration of chronic disease (6.3 ± 4.41 versus 7 ± 5 years; p > 0.05). However, children who received vaccine were younger than those who did not (mean age 8.2 ± 4.3 versus 12.5 ± 4 years, respectively; p < 0.0001). In 2008-2009, a total of 208 (61%) of the 343 children with chronic disease conditions were vaccinated for influenza and 135 (39%) were not. A very similar pattern was observed in the same population in 2007-2008. Similarly to findings for pneumococcus, vaccination rate for influenza was significantly lower in patients with DM compared with HIV (p = 0.01), CF (p < 0.0001) and LTx (p = 0.0006). Influenza vaccination rates in children seen in the 3 Reference Centres for DM were broad ranging from 21% to 61% (figure 1). Vaccinated children were younger than not vaccinated (mean age 11 ± 4.2 versus 12 ± 4.2 years, respectively; p = 0.03), but the mean duration of the chronic conditions was significantly higher in vaccinated subjects (7.8 ± 4.9 years) than in non vaccinated subjects (6.5 ± 5 years; p = 0.001). This apparently surprising result is explained by the lower age at diagnosis of children with HIV, CF and LTx compared to DM.

### Role of physicians working in different settings in recommending vaccinations

**Figure 2:**
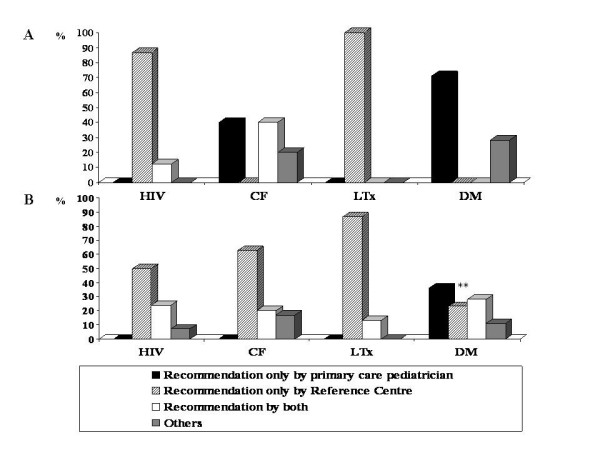
**Correlates of pneumococcal and influenza vaccinations in 343 high-risk children**. **A**. Reasons for receiving pneumococcal vaccination. Role of the Reference Centre in recommending vaccination in HIV vs CF and DM: p < 0.05. Role of the Reference Centre in recommending vaccination in LTx vs CF and DM: p < 0.05. **B**. Reasons for receiving influenza vaccination. ** DM versus HIV, CF and LTx: p < 0.0001. In both histograms, "others" includes vaccination because of previous serious influenza illness, recommendation by relatives or friends, information garnered from the mass media or not specified reason.

To investigate the role in promoting immunization by the physicians working in specific Reference Centres, key responders were asked to report who recommended vaccinations. As shown in figure 2A, physicians of the Reference Centres for HIV and LTx actively recommended immunization against pneumococcus, while this was not so in the 3 centres for DM. For CF patients, physicians of the Reference Centres and primary care paediatricians were both in charge of recommending pneumococcal vaccination. Again, the Reference Centre had a major role in recommending influenza vaccination to children with HIV, CF and LTx, whereas primary care paediatricians had a main role in case of children with DM (figure 2B).

### Reasons for missing vaccinations

**Figure 3:**
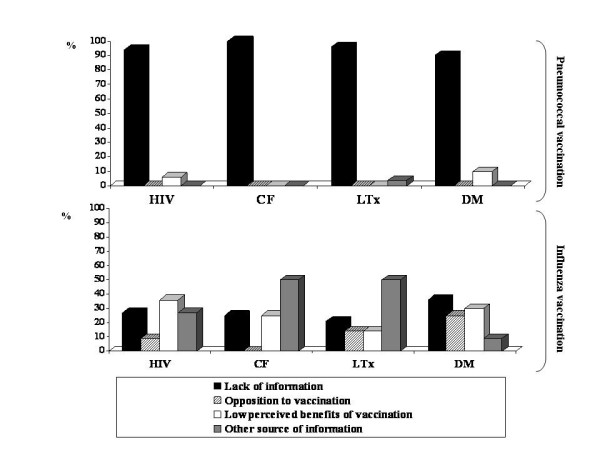
**Reasons for not having pneumococcal or influenza vaccinations in 343 high-risk children**. "Other source of information" includes child barriers ("child was sick"), parents barriers ("no time", "forgot"), system barriers ("no vaccine available"), vaccination considered unnecessary by physician or not specified reason.

There was a wide spectrum of reasons why patients were not vaccinated for pneumococcus and influenza, ranging from lack of information to parental "ideological" attitudes and concern (figure 3). Lack of information prevailed in all groups, particularly as regards pneumococcal vaccination.

Key informants were specifically asked whether they would consider pneumococcus and influenza infections as potentially serious diseases in their children. As a control question, we also asked whether they would consider these infections a severe problem in healthy children. Overall, a small number of caregivers (79/343, 23%) were aware of pneumococcus infection and this was perceived as a potentially severe infection by only 15% of them. The same percentage (53/343, 15%) of caregivers considered influenza as a serious infection, but an higher proportion (243/343, 71%) considered it a severe disease in presence of underlying chronic medical conditions. A greater proportion of caregivers of CF (36/39, 92%) and LTx patients (50/59, 85%) were concerned about influenza infection than caregivers of HIV (25/40, 62%; p = 0.002 and p = 0.01, respectively) and DM children (134/205, 65%; p = 0.0005 and p = 0.003, respectively).

Awareness of the severity of pneumococcus and influenza infections, as reported by caregivers, was then correlated with vaccination rates. Significantly more children of caregivers who were aware that pneumococcus infection could be severe, were vaccinated than children of caregivers who did not considered pneumococcus infection as severe (12% versus 32%; p < 0.0001). No relationship between infection awareness and vaccination rate was found for influenza.

### Health care providers' behaviours in recommending vaccinations

Physicians working in 5 of 6 the Reference Centres reported that they actively recommended immunization against pneumococcal and influenza to high-risk children as part of their routine approach to children with chronic conditions. Physicians working within the same Reference Centre had a similar vaccination policy. In one of the 3 Reference Centres for DM, paediatricians advised influenza vaccination only to patients with poor metabolic control, whereas vaccination was considered unnecessary and not recommended in other children with DM. Interestingly, only 20 (25%) of the 80 children with DM followed at that Centre had been immunised, thus supporting the role of Reference Centre in promoting or not immunization. A total of 113 primary care paediatricians were interviewed. The majority of them were recommended pneumococcal (102/113, 90%) and influenza (109/113, 96%) vaccinations for children with chronic medical conditions.

## Discussion

Vaccination against influenza of healthy children aged 6 months-18 years is recommended in the USA [8] but not in Italy [9]. However, vaccination of children with chronic medical conditions against a wide spectrum of infectious diseases has been included in the Essential Levels of Care and is provided free of charge [9].

Although it is well known that pneumococcal infection can be more severe than influenza and that pneumococcal vaccine is protective for a long time, the vaccination rate against pneumococcus was low in all studied groups and far lower than influenza. Data from the United States showed that coverage rate for pneumococcal polysaccharide vaccine was only 37% among persons 18-64 year of age at increased risk for pneumococcal diseases [17]. As for paediatric age, recent data reported an estimated coverage rate for pneumococcal conjugate vaccines of 31, 38 and 49% among children born in 2000, 2001 and 2002, respectively [18]. Approximately 60% of children with the selected chronic conditions were vaccinated against influenza. This immunization rate was stable over two subsequent seasons and was higher than that reported in previous studies [19-21].

A suboptimal vaccination rate in at-risk children may be in part related to a lack of information, fear of side effects of vaccination in children who are already ill, to organisational problems, difficulties to identify at-risk subjects and low priority by public health authorities. In our study, immunization rates were closely related to the underlying condition, with the lowest rate for both vaccinations occurring in patients with DM. This may be due to the lack of awareness of the risks related with pneumococcus and influenza infections by physicians in charge of children with DM.

Furthermore, for pneumococcal vaccination, "once in the life" administration instead of annual administration (as required by influenza vaccine) could reduce the opportunities that physicians have to check vaccination status. In the recent years, the Italian vaccination policy has actively promoted pneumococcal vaccination in all children aged less that 24 months [9]. This policy may explain the younger age of children vaccinated for pneumococcus, compared to non vaccinated, found in the present study. Another potential reason of the low coverage rate could be the lack of awareness of the severity of pneumococcal and influenza infections in at-risk children, as reported by caregivers of patients with HIV and DM.

Immunization includes three different actions: to recommend vaccination, to administer vaccine, to check vaccination status. It was previously found that one of the most important factors positively associated with vaccination was recommendation by a health care provider [22-24]. However, it has been reported that health care workers' knowledge about recommendations on vaccinations is limited [11,25], and, in a previous study on the determinants of influenza vaccination, the majority of parents of infants stated they received information through mass media rather than from physicians [23]. The main information that emerges from this study is that at-risk children depend from three categories of physicians: disease specialists, primary care physicians and vaccination service physicians. These categories are equally encharged with vaccination, with no clear distinction between them. This policy, characterized by a redundancy of physicians' roles, leads to an ineffective recommendation and check of vaccination status.

## Conclusions

Three major findings emerge from this study. Firstly, although both pneumococcal and influenza vaccines in Italy are provided free of charge, the immunization rate for pneumococcal infection is very low, whereas the immunization rate for influenza is relatively high, with a pattern consistent over time. Secondly, for most chronic conditions, vaccination is performed upon the advice of a Reference Centre. Third, the main reason of unvaccination was the lack of knowledge by parents of the benefits of pneumococcal and influenza vaccinations in children with chronic underlying diseases.

In conclusion, immunization should be considered part of the global care of children with chronic medical conditions. Considering that the lack of recommendation led to missed vaccination, providers need to be aware that a particular condition is an indications for influenza and/or pneumococcal vaccinations and should actively recommend and control vaccination. For diseases principally managed by Reference Centres (such as HIV, CF and LTx), physicians of the Reference Centres should mainly be in charge of vaccination policy. For diseases with a has a less defined management, this role should be played by primary care paediatricians.

To achieve optimal immunization rates in at-risk groups a complex array of responsibilities and functions is required. A special effort is required to implement pneumococcal immunization among Italian high-risk children.

## Competing interests

The authors declare that they have no competing interests.

## Authors' contributions

AnG had primary responsibility for project development, analyses of data and writing the manuscript. ALV and VS contributed to the analysis of data and drafting of the manuscript. MTR and AB contributed to the enrolment of patients and collection of data. AG conceived the article and approved the final draft of the paper.

All authors have read and approved the final version of the manuscript.
